# Talking about living and dying with the oldest old: public involvement in a study on end of life care in care homes

**DOI:** 10.1186/1472-684X-10-20

**Published:** 2011-11-23

**Authors:** Claire Goodman, Elspeth Mathie, Marion Cowe, Alex Mendoza, Daphne Westwood, Diane Munday, Patricia M Wilson, Clare Crang, Katherine Froggatt, Steve Iliffe, Jill Manthorpe, Heather Gage, Stephen Barclay

**Affiliations:** 1 Centre for Research in Primary and Community Care, University of Hertfordshire, College Lane, Hatfield, AL10 9AB UK; 2 The General Practice and Primary Care Research Unit, Department of Public Health and Primary Care, Institute of Public Health, Forvie Site, Robinson Way, Cambridge, CB2 0SR, UK; 3 School of Health and Medicine, Division of Health Research, Bowland Tower East, Lancaster University, LA1 4YT, UK; 4 Department of Primary Care and Population Health, University College London, Hampstead Campus, Rowland Hill St, London, NW3 2PF,UK; 5 Social Care Workforce Research Unit, Kings College London, King's College London, The Strand, London, WC2R 2LS, UK; 6 Department of Economics, University of Surrey, GU2 7XH, UK; 7 General Practice Research Unit, Institute of Public Health, University of Cambridge, Forvie Site, Robinson Way, Cambridge, CB2 0SR, UK

## Abstract

**Background:**

Public involvement in research on sensitive subjects, such as death and dying, can help to ensure that questions are framed to reflect the interests of their peers, develop a shared understanding of issues raised, and moderate the often unequal power relationship between researcher and participant. This paper describes the contribution and impact of older members of a Public Involvement in Research group (PIRg) to a study on living and dying in care homes.

**Methods:**

A longitudinal study, with a mixed method approach, its aims were to capture key experiences, events and change over one year, of older people resident in participating care homes in the East of England. Residents were interviewed up to three times and their case notes were reviewed four times over the year. Interviews were semi structured, and recorded. Four members of a Public Involvement in Research group (PIRg) contributed to preliminary discussions about the research and three were involved with many of the subsequent stages of the research process including the facilitation of discussion groups with residents.

**Results:**

There were three areas where the involvement of the Public Involvement in Research group (PIRg) positively influenced the study process. These were recruitment, governance and safeguarding, and in collaboration with the residents in the care homes, the discussion and interpretation of emergent findings. PIRg members were of similar age to the residents and their involvement provided different and often more reflective insights of the significance of the findings for the participants. There were examples where decision making about the range of PIRg participation was not always negotiable, and this raised issues about power relationships within the team. Nevertheless, PIRg members expressed personal benefit and satisfaction through participating in the research and a commitment to continue to support research with this older age group.

**Conclusions:**

The contribution of the PIRg supported a successful recruitment process that exceeded response rates of other studies in care homes. It safeguarded residents during the conduct of research on a sensitive topic and helped in validating the interview data gathered by the researchers through the discussion groups facilitated by the PIRg. There were power differentials that persisted and affected PIRg participation. The study has showed the value of developing job descriptions and a more formal means of setting out respective expectations. Future research may wish to elicit the views of focal participants in such studies about the mediation of research by public involvement in research.

## Introduction

Involving the public (patients, carers and the public) in health care research has been widely discussed and endorsed in English policy [1-3]. However, reviews of Patient and Public Involvement (PPI) impact and effectiveness suggest that there is a need to understand if different approaches to PPI achieve different outcomes [4-8]. In end of life care research there is some evidence that "bottom up" approaches focusing on value to the user may provide a framework for designing programmes to raise awareness of issues related to death and change behaviours [9]. This paper describes the involvement of members of a PPI group in a study that explored the views, expectations and experiences of living and dying of older people living in care homes. It considers how their involvement as co-researchers supported recruitment to a study on a sensitive topic, provided peer support and safeguarding for vulnerable participants, and informed how findings were interpreted both with the research team and the staff and residents of the care homes. It also discusses what the process revealed about power relationships within the research team and how public involvement is negotiated.

## Background

The involvement of the public in research with older people builds upon the wider work of peer facilitation/peer education where people respond well and identify with those in a similar situation or of a similar age [10]. The rise in user involvement in research is due to the emergence of participatory research that seeks to work 'with' people rather than 'on' them as subjects of research, political drivers for greater user participation, policies that reflects broader consumerist and democratic movements within society [11,12]. It can involve consultation, collaboration or be user-led/controlled [13,14]. Studies that have used PPI to focus on how older people anticipate end of life and their priorities for care have involved older people acting as peer educators whose role is to share information with people of a similar age and life experience [15-17]. PPI involvement in research on sensitive subjects, such as death and dying, can ensure that questions are framed to reflect the interests of their peers, develop a shared understanding of issues raised, and moderate the often unequal power relationship between researcher and participant [18]. This paper describes the involvement of older people as co-researchers in a study of living and dying in care homes. It considers the advantages and disadvantages of user participation in end of life and care home research

## The EPOCH Study

The Experiences of Older People in Care Homes (EPOCH) study aimed to understand how living in a care home influences, over time, residents' views, experiences and expectations of end of life care and symptom relief. To capture key experiences, events and change over one year, of older people resident in the participating care homes, a longitudinal, mixed method design was employed. Residents were interviewed up to three times and their case notes were reviewed four times over the year. Interviews were semi structured, recorded and fully transcribed. Issues and themes identified in the first interview were discussed in subsequent interviews to see if key events, or the passage of time, had affected how older people thought and talked about living and dying in a care home. Information about the deaths that occurred during the study was reviewed and an economic analysis of their prior service utilisation was carried out. Care home and health care staff (General Practitioners, Community and palliative care nurses) were interviewed once about their experience and views of providing end of life care and discussion groups were held with residents to discuss emergent findings towards the end of the year's data collection ([19] provides a detailed account of the study method).

### Organisation of PPI

Patient and Public involvement (PPI) may occur at all stages of the research process [3,20]. In this study PPI was provided by four members of a Public Involvement in Research group (PIRg) that was linked to the university where the research was based. The PIRg members had completed an eight day introduction to research methods course as well as specific training on discussing end of life issues led by an academic (Dr Amanda Clarke) with previous experience of leading studies where older people facilitated data collection on end of life [15,21-23]. PIRg members and researchers met regularly and completed reflective diaries after each visit to a care home.

The PIRg members contributed to preliminary discussions about the research and three were involved with subsequent stages. This included visiting the care homes with the researchers, explaining the study to residents and their family members, and facilitating discussion groups with residents. Their ages ranged from 75-81, three members had direct experience of close relatives and friends living and dying in a care home. This paper focuses on the contribution of the three PIRg members who were involved in recruitment, review of study documentation, analysis, discussion and feedback of preliminary findings to care home residents. Table 1 provides a summary of the range and detail of their involvement.

**Table 1:** Public involvement in different stages of research process

PIR Involvement	Level of Involvement
Study Design	Input on grant proposal and development of method for public involvement within the study

Steering Committee	Regular attendance

Review of study documentation	Information leaflets, consent forms Interview guides (residents)

Recruitment	Explanation of information leaflet and consenting process to care home residents

Data analysis	Reading transcripts, identification of key themes. Meeting to discuss findings/research team Running discussion groups with care home residents to discuss emergent themes

Report/Dissemination	Commenting on final report. Report to funders. Co-presentation and sole presenters at National Care Home Forum

The PIRg members had honorary contracts with the University and had been subject to Criminal Record Bureau (CRB) checks (as required by many health care facilities for researchers in the UK). They received an annual honorarium, study related expenses and financial support to attend conferences. PIRg members and researchers met at the beginning of the study to talk about how the study might affect them, e.g. distressing conversations with care home residents, talking with people with dementia, residents dying and their own motivation for involvement. Regular meetings were held throughout the study. They were also encouraged to complete reflective diaries (as were the researchers) after each visit to a care home.

The study was reviewed by the Southampton and South West ethics committee, Reference: 08/H0502/38.

## Impact of Public involvement

The research study recruited 121 (47%) of the residents from 6 care homes (with no on site nursing provision) in three geographically disparate areas in England. Thirty care home staff and 19 NHS staff were also interviewed. Sixty three older people participated in up to three interviews over the year's data collection of which 23 (5%) of the residents died during the year. A detailed account of the study findings is provided in the final report [24].

The three PIRg members spent between 2-6 hours in the care home during each visit, and a total of approximately 80 hours in care homes over the course of the study. They made between 6 and 18 individual visits to the care homes. The PIRg members visited the care homes mostly at the beginning and end of the study. The PIRg members and researchers spent similar amounts of time in the care homes during the consent and recruitment phase. The researchers carried out all the data collection (interviews, care note reviews) and towards the end the PIRg members (accompanied by researchers) discussed the emergent findings with residents.

There were three areas where PPI involvement directly influenced the outcomes of the study. These were recruitment, governance and safeguarding, and the discussion and interpretation of emergent findings with the older people living in the care homes.

### PPI as collaborators in the recruitment process

Recruitment to the study was a staged process, that involved securing care home manager agreement to participate, informal gatherings in the care home to explain the study (e.g. coffee mornings and residents' and relatives' meetings) and conversations with individual residents about possible participation supported by study documentation. All the participant care homes were interested in improving how they provided end of life care for their residents. Interviews with care home staff demonstrated that it was an area of care they found difficult and because of this the majority of staff were willing to support the recruitment process and the involvement of PIRg members. The PIRg members accompanied the researchers to introduce the study to the residents of the care home. To maximise opportunities for participation, sufficient time was allowed and careful attention paid to the particular needs of people with sensory impairments, and those who may have had difficulty communicating. The PIRg members had contributed to the development of the information leaflets; however, it was often necessary to explain further the lengthy information leaflet (as required for research ethics approval and governance) and the consent process. PIRg members answered questions and discussed with residents what the possible advantages and disadvantages of participation might be. These discussions about the study were independent of the process of obtaining consent that the research fellows undertook.

### PPI as a safeguard and support to research governance

There was a risk that participants could feel obliged to participate, or become distressed when talking about what had led to them moving to a care home or being asked about their future. PIRg members had a key role in helping to ensure that these risks were acknowledged, that residents who expressed doubts about participation were supported in their decision making and that the team had the capacity to address any unintended consequences arising from inviting people to participate in the study and in the interviews themselves.

During the year of data collection 156 interviews were completed with 63 residents. Originally, it had been intended to involve PIRg members in the interviews; however it was not possible to ensure that the same PIRg member could maintain the continuity of involvement required for the three interviews over the year of data collection. Instead, PIRg members accompanied the researchers to the care homes to help explain the study, sat with residents and helped to minimize the impact of the study on the care home staff by ensuring that they were not taken away from care work when residents wanted to continue talking once an interview had finished. Participation in the study could at times be an emotional experience for residents. On these occasions if an interview was stopped or time was needed after the interview to reflect on the issues raised, PIRg involvement increased the capacity within the research team to address this. On one occasion a participant did not want to talk further but was visibly upset. He had talked with a PIRg member during the recruitment phase of the study and she sat with him, and stayed till he was more composed and, with his permission, told a care worker that the interview had evoked distressing memories. For the majority of the participants however, the staged interviews offered a valued opportunity to talk about living and anticipating dying in a care home and enabled them to build a rapport with members of the research team. PIRg involvement was also a source of support to the research team in what was often emotionally demanding work. For example; one PIRg member whose partner had had dementia provided support to a member of the research team who had had limited experience of talking to people living with dementia.

PIRg members were able to 'fit' into the care homes, talk comfortably with the residents and make sure the residents fully engaged with the consent process. They had the time to explain the study, repeating the necessary information and reinforce key messages that there was no obligation to participate.

### Involvement in the development of the research and interpretation of findings

The PIRg members acted as a 'critical friend' to the researchers (questioning assumptions and the research processes). It was a particular challenge to develop information materials that made clear that the focus of the study was the anticipation of dying. PIRg members reviewed all the study support materials (information sheets and topic guides) prior to their submission for ethical review to ensure that information was accessible and unambiguous.

The PIRg members were active discussants in the analysis of the findings, reading through interview transcipts, annotating their reactions and thoughts to what was being said and identifying themes across the interviews and across the care homes; in meetings with the research team these informed the themes developed in the qualitative analysis (undertaken with the data software programme NVIVO). PIRg members also assisted with comments and field notes (in their reflective diaries) on the culture and ambiance of the care homes and commented on the draft final report, and conference presentations.

An unanticipated benefit of PIRg involvement was that they were also a source of continuity working with different researchers at different sites. This meant they could share learning about optimum times to visit the sites and anticipate questions and issues that different participants had raised about the study.

Towards the end of the study, discussion groups were held with residents in the care homes. The purpose was twofold. To discuss emergent findings with residents, exploring to what extent participants recognised and could relate to key themes, and secondly, to consider the impact of participation in the study. The PIRg members helped to develop the discussion guide for these groups and two PIRg members facilitated each group supported by a researcher who acted as note taker. The PIRg members reported back the main findings to the group in their own words and asked residents to comment or further expand. They also shared their own feelings about how they anticipated dying and their responses to the study itself. This approach encouraged residents unfamiliar with a group discussion format to react to statements and to talk about the research openly, possibly because they were discussing their involvement with a third party. PIRg members were of similar age to the residents. The residents joked with some of the PIRg members that they would soon be in the care home themselves. Their involvement appeared to offer different perspective to that created between the researcher/resident, encourage questions in an environment where there were few opportunities for shared discussion.

The focus groups confirmed findings from the interviews such as; wanting more information about fellow residents who had died, and a general feeling that they lacked the opportunity to talk in-depth with anyone at the care homes about subjects that were important to them (not just their views and priorities for the future).

At the dissemination phase, members of the group were co presenters and sole presenters in seminars and conferences to primary care health professionals as well as practitioners and researchers working with and in care homes.

### Impact and level of participation on the PIR group members

One PIRg member who had volunteered to visit the care homes, joined the project steering group instead. This was because there were concerns from the research team that her espoused views about assisted care for dying people could be in conflict with the exploratory approach of the study. This member felt she could have made a greater contribution if involved in the care home visits, and this tension highlighted the power relationship between researchers and members of the PIRg. While PIRg members were involved in every stage of the study, from the development of the research proposal to the dissemination and discussion of findings, decision making about the boundaries and range of PIRg participation were not always negotiable. Something that only became apparent once the study began.

Other PIRg members reported personal benefit from participating in the study and expressed a desire to continue to support research with this older age group. Their experiences while taking part in the research strengthened the skill base of the PIRg at the University and provided new insights into the research process.

## Discussion

It would be misleading to suggest the public involvement in end of life research brings unalloyed benefit. It was time consuming and resource intensive to liaise with members and careful planning was necessary to prepare for PIRg participation and ensure there was a common understanding of the study purpose, the contribution of different members of the team and the particular sensitivities of talking about dying.

For the research team, research deadlines, adherence to the protocol as funded, and responsibility for the research study as a whole meant that PIRg members were not always treated as equal partners. The project was situated within the participatory as opposed to the emancipatory paradigm and hence the overall control remained with the researchers [25,26]. Many researchers feel more comfortable in retaining control and working within a professionally defined construction of public involvement [27]. Beresford [11,28] argues that there is an innate fear on the part of many researchers that service user and public involvement may reduce the rigour and reliability of research findings. While the researchers involved in this project had a strong commitment to public involvement in research, there were preoccupation about the impact of potential co-researchers' values on the research outcome that were not applied to other members of the research team. This perceived/presumed dichotomy between co-researcher as value-laden and emotive whilst the researcher is objective and unbiased is not helpful, not least because the researchers bring their own set of values and emotions to a study [29,30]. The PIRg member who was excluded from visiting the care homes, considered she was as capable of being objective and unbiased about the methodology and findings as the researchers.

Within participatory research negotiation between the researchers and service users or the public can be one of the most challenging issues to face but there is little research on this aspect [31]. One practical way of addressing this is through a formalised agreement about roles and expectations [32]. In the EPOCH study there was no formal job description for the PIRg members and there was a risk that despite the honorarium and benefits in kind that PIRg members received, the study exploited their good will and interest. It was difficult to know how much time to ask a member to give to the project. These issues highlights the value of project specific role specification for PIRg involvement, the importance of regular review, and the need to develop open working relationships prior to a study's commencement.

There were also logistical challenges. PIRg members were available for varying amounts of time due to personal and other commitments. The researchers sometimes found it difficult to accommodate their needs; because care home visits could not always be organised with sufficient notice or could be cancelled by the care home manager with minimal warning.

This was an emotionally demanding study. The researchers and PIRg members agreed to complete a reflective diary after each visit to a care home, although, not all members did this. Mechanisms had been put in place to support the research team and the PIRg members, there were debriefing meetings to discuss areas of concern and matters that the research team had found difficult to address and PIRg members could access extra support independent to the study if wanted. Participation triggered memories of family members who had died and whose experience of care homes had not always been good. For two PIRg members a desire to make a difference in this area of practice had been a key motivation for their involvement.

The study was essentially exploratory, and while recommendations for future practice were based on the findings, the study itself did not result in any immediate changes in patterns of care or service provision. It was a source of frustration, for some members of the PIRg and research team that research may not directly influence practice in care homes when it was apparent (to them) that change was needed. In contrast one PIRg member considered the research experience had given her hope and challenged previous bad experiences of care home life. The care homes in this study had staff who were interested in improving the end of life care they provided.

## Conclusions

This paper examined some of the practical implications of having PIRg members involved in end of life research in care homes. Their involvement included but extended beyond involvement at a distance in the review of research questions, documents and findings.

The recruitment period required considerable time and input from the researchers and from PIRg members resulting in a successful recruitment process that exceeded response rates of other studies in care homes [33], while also safeguarding residents during the conduct of research on potentially sensitive topics. There were power differentials that, despite the research team's efforts in involving the PIRg throughout the research process, persisted. It has also illuminated how the evidence created in a project is as much shaped by social relationships in the research process as the actual methodologies [34].

Future studies would benefit from developing a job description for PIRg members and a more formal means of setting out respective expectations. Future research may wish to elicit the views of focal participants in such studies and other stakeholders about the mediation of research by PIRg members. In this study these groups would include residents but also their family members and the staff of the care homes.

## Completing interests

The authors declare that they have no competing interests.

## Authors' contributions

CG designed the study with KF, SB, HG, SI, JM and drafted the paper, MC, AM, DM, DW were the PIR members EJM, CC, led the data collection and analysis, PMW is the lead of the PIR group. All authors contributed to the paper, reviewed drafts and approved the final content.

## Pre-publication history

The pre-publication history for this paper can be accessed here:

http://www.biomedcentral.com/1472-684X/10/20/prepub
